# Widespread distribution of hepatitis E virus in Spanish pig herds

**DOI:** 10.1186/1756-0500-4-412

**Published:** 2011-10-14

**Authors:** Nereida Jiménez de Oya, Ignacio de Blas, Ana-Belén Blázquez, Miguel A Martín-Acebes, Nabil Halaihel, Olivia Gironés, Juan-Carlos Saiz, Estela Escribano-Romero

**Affiliations:** 1 Department of Biotechnology. INIA. Madrid, Spain; 2 Department of Animal Pathology. Faculty of Veterinary. Saragossa University, Spain

## Abstract

**Background:**

Hepatitis E virus (HEV) infection is a serious health problem in developing countries and is also increasingly reported in industrialized regions. HEV is considered a zoonotic agent and strains isolated from swine and human sources are genetically similar. Thus, HEV is of increasing importance to both public and animal health. The aim of the present study was to evaluate the distribution of HEV in a large population of pigs from herds located in different autonomous regions throughout Spain.

**Results:**

The presence of anti-HEV IgG antibodies was analyzed in 1141 swine serum samples (corresponding to 381 pigs younger than 6 months and 760 pigs older than 6 months) collected from 85 herds. Herds were located in 6 provinces in 4 autonomous regions throughout Spain. At least one pig tested positive for anti-HEV IgG in over 80% of herds. Of individual pigs, 20.4% (233/1141) were positive for anti-HEV IgG, with the prevalence being higher in adult pigs than in those under 6 months (30.2% *vs. *15.5%). A subset of serum samples taken at 2- to 5-week intervals showed that seroprevalence dropped between 3 and 11 weeks of age, and then rose significantly by the 15th week. Pigs were also examined for the presence of HEV-RNA by RT-PCR. Of pigs tested for the presence of HEV-RNA 18.8% (64/341) were positive, with at least one pig in almost half of the herds testing positive. HEV-RNA amplicons from several positive pigs were sequenced and all were of genotype 3.

**Conclusions:**

HEV was found to be widely distributed among swine farms across Spain, with the prevalence being highest among animals older than 6 months. These results indicate that HEV infection either is or is likely to become endemic in the Spanish swine population.

## Background

Hepatitis E is an enterically transmitted disease caused by the hepatitis E virus (HEV). It was first detected in tropical and subtropical areas with poor sanitary conditions and inadequate water supplies, where it is considered endemic [1-3]. Although only a single serotype is recognized, considerable genetic diversity has been observed among HEV isolates, which are grouped into four genotypes (gt). Viruses responsible for epidemics and sporadic cases in endemic regions are mainly classified into gt 1 and 2, and those detected in sporadic cases of acute hepatitis E elsewhere in the world are classified into gt 3 and 4 [1-3].

In non-endemic regions, where outbreaks have not been described, the disease accounts for only a minority of reported cases of acute viral hepatitis. Until recently, most of these cases were considered to be imported by travellers to HEV-endemic areas. However, in recent years, an increasing number of sporadic cases related to autochthonous transmission of HEV have been recorded in the USA, Europe, and developed countries of the Asia-Pacific region, in all of which it is considered an emerging disease [4]. In contrast to what is seen in endemic areas, these indigenous infections are mainly caused by gt 3 strains.

Pigs are a recognized reservoir for HEV [1,5,6] and a possible source of HEV transmission to human beings [1-4,7], highlighting the potential of HEV as a zoonotic pathogen. Indeed, HEV seems to be an enzootic infection in pigs as shown by the high prevalence rates of anti-HEV antibodies and HEV-RNA detected in them. Furthermore, human and pig HEV sequences, especially those from nearby geographical areas, have been found to be quite similar or even identical [8]. Recently, several studies have described a relationship between HEV infection and occupational exposure to pigs, such as in farmers, veterinarians, butchers, or slaughterhouse workers, in which the number of acute hepatitis E cases and seroprevalence rates seems to be higher than in non-exposed populations [1,9,10]. In addition, human HEV infection via the consumption of contaminated pork products has also been reported [11,12].

Since the first detection of HEV in pigs in 1997 [13], the virus has been identified on swine farms from many geographical areas, including Europe. However, the reported prevalence has been quite variable, from 22% to 55% [1,14-16], and although pigs aged 2-4 months seem to be more susceptible to the infection [15,17-19], HEV RNA has also been detected in finishers and breeders [20].

Porcine production in Spain, with more than 25 million pigs, is the second highest in the European Union with an annual economic impact in excess of 4 million Euros [21]. There is evidence of the presence of HEV in the country in what seems to be some autochthonous human cases [22-25], as well as in wild boars [20,26] and pigs [20], in which the virus has been present since at least 1985 [27].

In this study, the presence of both anti-HEV antibodies and HEV-RNA has been analyzed in a large population of pigs from herds located in different autonomous regions across Spain, establishing that the virus is indeed distributed among the Spanish swine population.

## Results

### Distribution of HEV in Spain

**Figure 1:**
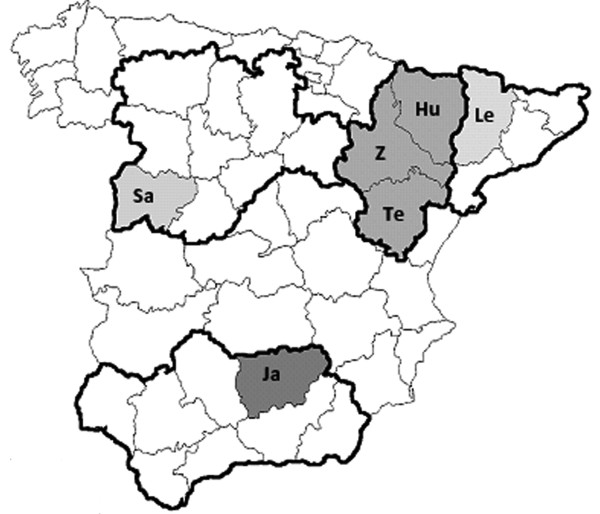
**Map of Spain showing the autonomous regions (broad outline) and provinces where sampling was conducted**. Hu: Huesca, Z: Saragossa, Te: Teruel, Le: Lleida, Ja: Jaen, Sa: Salamanca.

A total of 233 of the 1141 (20.4%) serum samples tested resulted positive for anti-HEV IgG (Table 1). There were seropositive adult and young pigs in all Spanish autonomous regions and provinces covered by the study. Analysis of the seroprevalence by province revealed a significantly unequal distribution, ranging between 2.5% and 26.4%, and these differences were statistically significant (p < 0.001). However, no geographical relationship was found regarding the seroprevalence rates. For instance, neighbouring provinces such as Lerida and Huesca (Figure 1) showed uneven seroprevalences, and similar patterns were also observed in farms located within a single province (data not shown).

**Table 1:** Seroprevalence of anti-HEV IgG in pigs in the provinces considered in the study

	Percentage (positive/total) of anti-HEV IgG							
	Province							
	Huesca	Saragossa	Teruel	Salamanca	Jaen	Lerida	All Provinces	p
Adult pigs	19.2% (5/26)	37.0% (95/257)	28.6% (8/28)	4.8% (2/42)	21.4% (3/14)	14.3% (2/14)	30.2% (115/381)	<0.001 *

Young pigs	18.2% (20/110)	19.2% (73/380)	12.0% (12/100)	1.3% (1/80)	20.0% (12/60)	0.0% (0/30)	15.5% (118/760)	<0.001 *

All Pigs	18.4% (25/136)	26.4% (168/637)	15.6% (20/128)	2.5% (3/122)	20.3% (15/74)	4.5% (2/44)	20.4% (233/1141)	<0.001 *

p	0.548 ^b^	<0.001 *	0.042 ^†^	0.272 ^†^	0,580 ^†^	0.096 ^†^	<0.001 *	

* Significance of Chi-square test; ^† ^Significance of Fisher's exact test

At least one pig tested positive for anti-HEV IgG in most of the herds tested (81.2%, 69/85), the percentage of positive farms varying between 79.2% and 100% among provinces (data not shown). Likewise, the proportion of tested pigs with anti-HEV IgG among positive farms was also quite variable, from 3% to 100%.

### Seroprevalence analysis by age

A statistically significantly higher proportion of positive animals was found in adult pigs than in those under 6 months of age (30.2% *vs. *15.5%, p < 0.001) (Table 1). The percentage of positive adults and young pigs varied from 4.8% to 37% and from 0% to 20%, respectively, depending on the province studied, but it was always higher in adults, these differences being statistically significant in animals from Teruel and Saragossa provinces.

Samples were taken from young swine from 13 herds from four autonomous regions at 2 to 5 week intervals. As shown in Table 2, the prevalence of anti-HEV IgG varies significantly (p < 0.001). The prevalence of positive pigs dropped from age 3 to 11 weeks (6.9% to 1.4%) and then rose significantly by the 15^th ^week (31.0%), then attaining values similar to those found overall (30.2%) in the adult swine population studied (Table 1).

**Table 2:** Seroprevalence of anti-HEV IgG in pigs at several week intervals

Percentage (positive/total) of anti-HEV IgG						
Age (weeks)						
3	5	7	11	15	20	All

6.9% (9/130)	3.3% (4/120)	1.7% (2/120)	1.4% (1/70)	31.0% (31/100)	17.5% (14/80)	9.8% (61/620)

Significance of Chi-square test, p < 0.001

### Presence of HEV-RNA in sera

341 serum samples from 72 herds from Aragon (30 farrow-to-finish and 42 grower/fattener farms) were also examined for the presence of HEV-RNA by RT-PCR (Table 3). A total of 64 pigs (18.8%) were HEV-RNA positive, the proportion of positive animals being significantly higher among pigs younger than 6 months of age than among those older than 6 months (25.7% vs. 13.9%, p = 0.006). At least one pig in almost half of the herds (47.2%) tested positive for HEV-RNA, with more positive pigs on farrow-to-finish than on grower/fattener farms (60% vs. 38.1%), although in this case the difference was not statistically significant (p = 0.066).

**Table 3:** Prevalence of HEV-RNA in individual pigs (older and younger than 6 months) and herds

	Group	Percentage prevalence (positive/total)	p*
Pigs	Adult	13.9% (28/201)	0.006
	Young	25.7% (36/140)	
	All	18.8% (64/341)	

Herds	Grower/Fattener	38.1% (16/42)	0.066
	Farrow-to-finish	60.0% (18/30)	
	All herds	47.2% (34/72)	

* Significance of Chi-square test

When HEV-RNA and anti-HEV IgG data were combined in the aforementioned subset of sera samples (Table 4), it was shown that almost half of the pigs (48.4%) did not present any marker of HEV infection. In 12% of them HEV-RNA, but not anti-HEV IgG, was detected; whilst both markers were present in 6.7% of the animals. Finally, evidence of a past infection was observed in 32.8% of the animals, since no HEV-RNA could be detected but they had anti-HEV IgG in sera.

**Table 4:** Prevalence of anti-HEV IgG and HEV-RNA in swine (older and younger than 6 months)

Percentage (positive/total)			
	Adult (201)	Young (140)	All pigs (341)
RNA-/IgG-	52.7% (106)	42.1% (59)	48.4% (165)
RNA+/IgG-	8.5% (17)	17.1% (24)	12.0% (41)
RNA-/IgG+	33.3% (67)	32.1% (45)	32.8% (112)
RNA+/IgG+	5.5% (11)	8.6% (12)	6.7% (23)

Significance of Chi-square test, p = 0.040

### Phylogenetic analyses

**Figure 2:**
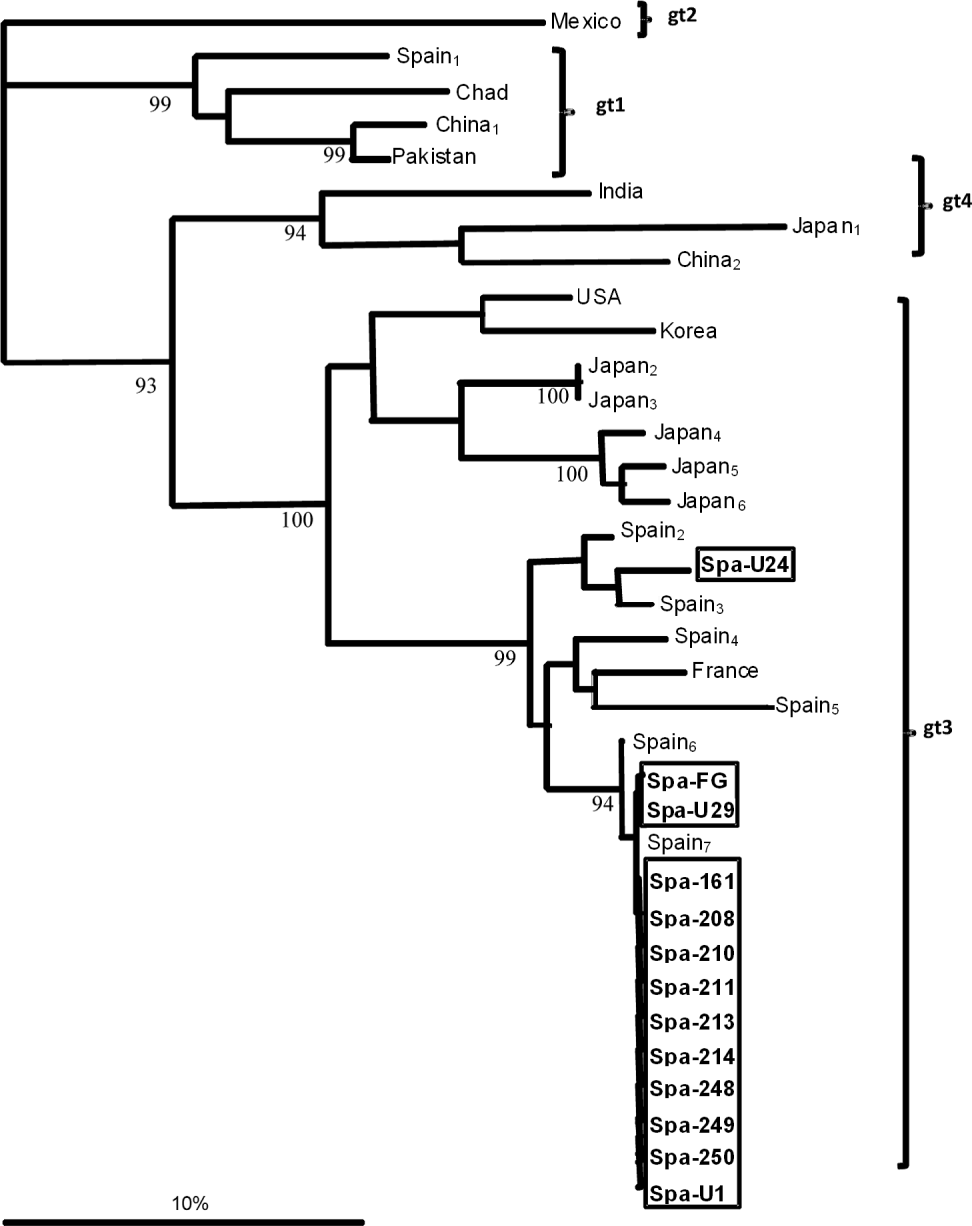
**Phylogenetic tree of the amplified HEV sequences**. The tree was constructed as described in the Methods with sequences from this study (in boxes) and sequences representing the four HEV gts (1-4) retrieved from GenBank (see Methods). Branch lengths are drawn to scale. The scale bar corresponds to 10% nucleotide sequence divergence. The number represents bootstrap proportions in support of the adjacent node, based on 100 resampling iterations. Only bootstrap proportions greater than 90% are shown.

Phylogenetic analyses showed that all sequenced samples belong to gt 3 (Figure 2). Genetic distance between them varied from 0% to 8%, which is a similar range to those in previously reported gt 3 European (0-9%) and Asian (13-21%) sequences, but lower than that found when compared with strains from gt 1, 2 or 4 (23-33%).

## Discussion

The prevalence of anti-HEV antibodies detected in the large number of pigs from throughout Spain analyzed in this report (20.4%, 233/1441) confirms the findings of previous studies conducted in a more limited number of animals across Europe that had suggested a widespread distribution of the virus among the swine population of the continent [15,28,29]. Furthermore, no significant differences were found between the proportions of positive animals found in the different regions analyzed, irrespective of the number of pigs raised there (Table 1). Although previous studies of a limited number of pigs carried out in the north-east of Spain [27,30] have reported slightly higher figures, it should be noted that a very wide distribution of HEV in swine herds has been reported around the world [1].

At least one pig tested positive for anti-HEV IgG in over 80% (69/85) of herds, although the prevalence was again quite variable between farms and locations, as reported before in other countries [1]. These differences could be due, at least partially, to the different farming structure of pig production, the routine management and hygiene practices applied on these farms, as well as the methods used in the different reports.

The proportion of anti-HEV IgG positive animals was always higher among adults (30.2%) than in those under 6 months of age (15.5%) in all the provinces considered. Further studies of the presence of specific antibodies in sera collected at different weeks of age in 13 farms located in different provinces across the country showed that 6.9% of the tested animals were positive by age three weeks, with only 1.4% positive at 11 weeks, rising to 30% at 15 weeks. These data are in line with those recently reported from other studies [31,32] and are consistent with the natural infection of pigs aged around 10 to 12 weeks [1]. Maternal antibodies to HEV might persist up to 9 weeks of age [1] and confer resistance to viral infection in young pigs, which may explain the decrease in the number of animals with specific IgG found here 3 weeks after birth. On the other hand, the observed increase in the number of IgG positive animals older than 3 months probably reflects how the proportion of pigs with positive serology rises once maternal immunity fades away. This conclusion is also supported by the higher proportion of pigs younger than 6 weeks of age in which HEV-RNA was detected by RT-PCR, as previously observed [1,14,15,30].

Of pigs tested for the presence of HEV-RNA, 18.8% (64/341) were positive, with at least one pig in almost half of the herds testing positive, indicating that HEV infection is indeed widespread among herds throughout Spain. Consistent with the above findings, HEV-RNA was more frequently detected in farms growing pigs younger than 6 weeks of age; however, the proportion of positive pigs in individual farms was again variable.

Almost half of the 341 pigs tested did not present anti-HEV IgG or HEV-RNA in sera, whilst both markers were detected in 6.7% of the cases. This could be due to either a recent infection, a re-infection caused by a short protective immunity, or even to the establishment of a persistent infection [31,33].

A significantly higher proportion of HEV-RNA positive pigs was found among young pigs (25.7% vs. 13.9%) and all sequenced samples belonged to gt3, confirming that HEV infection is more frequent in young swine and that gt3 is highly prevalent in European pigs [1].

On the other hand, the data reported here imply that adult animals also become infected by HEV [31], which therefore means that infected pigs can enter into the slaughterhouse and thereby the food-chain. In fact, HEV-RNA has been detected in raw pig livers sold in local markets in the Netherlands, U.S.A., Korea, India and Japan [12,34-37] and infection of slaughterhouse workers has also been reported recently [24]. Infected pigs have a transient viremia lasting 1-2 weeks and shed the virus in faeces for about 3-7 weeks, making this a very important issue, since food quality and human health in general may be affected.

## Conclusions

In conclusion, the results obtained here in a large number of animals show that HEV is widely distributed among swine farms (over 80% had at least one anti-HEV IgG positive animal) and pigs (20.4%, had anti-HEV IgG in sera) throughout Spain, there being a higher prevalence among animals older than 6 months. In addition, HEV-RNA was detected in 18.8% of the tested sera and in almost half of the herds, confirming that the infection is currently active in the swine population These results indicate that HEV infection either already is, or is likely to become, endemic in the Spanish swine population.

## Methods

### Samples

As part of a large project carried out in Spain to assess the prevalence of different porcine pathogens, 1141 randomly selected swine serum samples, 760 from adults (aged 6 months or more) and 381 from young pigs (aged less than 6 months), were included in this study. Samples (14 per farm, on average) were collected from 85 pig farms located in six provinces in four autonomous regions of Spain (Figure 1). Two of them, Aragon and Catalonia, raise a high proportion of the Spanish swine population, 21.8% and 26.2%, respectively, while in the other two (Castile and Leon and Andalusia) the swine population is much lower, 5.7% and 8.3% of the total, respectively [21]. In three regions (Castile and Leon, Andalusia and Catalonia) all farms were located in the same province (Salamanca, Jaen and Lerida, respectively); while in Aragon farms were located in three different provinces (Huesca, Saragossa and Teruel). Additionally, in a subset of 620 young pigs from 13 of these farms, located in the four autonomous regions, blood samples were taken at week intervals (3, 5, 7, 11, 15 and 20 weeks of age). Random samples (7 to 10 animals per sampling time point) were collected, so that although they may not have corresponded to the same animals, they always came from animals from the same farm. Experiments were approved and performed according to the guidelines for animal experimentation of the Animal Safety Committee of our Institution.

### Assessment of HEV infection

Serum samples were tested for the presence of specific anti-HEV IgG antibodies by means of an ELISA based on a recombinant HEV gt 3 ORF-2 antigen expressed in *Trichoplusia ni *larvae that was shown to be highly specific and sensitive when compared with a widely used commercial kit [38,39]. Detection of HEV-RNA was conducted on 341 serum samples from 201 adults and 140 young pigs from 72 herds. To this purpose, total RNA was extracted from 140 μl of swine sera using QIAamp^®^Viral RNA mini kit (Qiagen, Valencia, CA). Nested reverse transcription PCR (nested-RT-PCR) was performed with 10 μl of isolated RNA using a commercial amplification kit (SuperScript™ One-Step RT-PCR, Invitrogen, Carlsbad, CA) as previously described [13], with outer primers 3156F (AATTATGCCCAGTACCGGGTTG) and 3157R2 (TCATAGTCTTGTATAACCACACG), and inner primers 3158F2 (TGGTCATGCTTTGTATTCATGG) and 3159R (AGCCGACGAAATCAATTCTGTC). Amplified products of 349 bp were identified by electrophoresis in a 2% agarose gel stained with ethidium bromide. An HEV-RNA positive sample [20], kindly provided by Dr. M.T. Pérez-Gracia, U. Cardenal Herrera-CEU, Valencia, Spain, was always included in the assays.

### Sequence and phylogenetic analyses

Amplicons from 13 randomly selected HEV-RNA positive samples were obtained as described above, purified and bidirectionally sequenced using nested forward and reverse oligonucleotides. The obtained sequences [GenBank: HQ148726-HQ148738] and some representative sequences of the four HEV genotypes [GenBank: M74506 (Mexico); AF058684 (Spain_1_); AY204877 (Chad); NC_001434 (China_1_); M80581 (Pakistan); AY723745 (India); AB099347 (Japan_1_); FJ610232 (China_2_); AF082843 (USA); FJ426404 (Korea); AB189070 (Japan_2_); AB189071 (Japan_3_); AB291955 (Japan_4_); AB291960 (Japan_5_); AB443627 (Japan_6_); DQ093564 (Spain_2_); DQ093567 (Spain_3_); EF523421 (Spain_4_); EU495148 (France); DQ093568 (Spain_5_); DQ093566 (Spain_6_); DQ093565 (Spain_7_)] where aligned and compared with the CLUSTAL W 1.6 program [40]. A phylogenetic tree was computed with the PHYLIP package [41].

### Statistical and epidemiological analyses

Prevalence was calculated as apparent prevalence, i.e., the ratio of positive results to the total number of samples. Proportions were compared using Chi-square test (or Fisher's exact test when appropiate). Statistical analyses were carried out with SPSS 15.0 for Windows and WinEpi http://www.winepi.net.

## Competing interests

The authors declare that they have no competing interests.

## Authors' contributions

NJ carried out most of the immunoassays and analysis of the results, participated in RNA extractions/amplification and helped to draft the manuscript. NH contributed to the molecular detection and the phylogenetic analysis. IB performed the statistical analysis. OG helped coordinate the sera collection, design the study and draft the manuscript. ABB conducted RNA extractions and amplification. MAMA participated in the serological detection by means of the immunoassay, and in the statistical analysis. JCS conceived the study, participated in its design and coordination and helped draft the manuscript. EER participated in the design of the study, helped in the immunoassays, coordinated the analysis of the data and drafted the manuscript. All authors read and approved the final version of the manuscript.
